# New version of the Pediatric Evaluation of Disability Inventory (PEDI-CAT): translation, cultural adaptation to Brazil and analyses of psychometric properties

**DOI:** 10.1590/bjpt-rbf.2014.0166

**Published:** 2016-06-16

**Authors:** Marisa C. Mancini, Wendy J. Coster, Maíra F. Amaral, Bruna S. Avelar, Raphael Freitas, Rosana F. Sampaio

**Affiliations:** 1 Programa de Pós-graduação em Ciências da Reabilitação, Escola de Educação Física, Fisioterapia e Terapia Ocupacional, Universidade Federal de Minas Gerais (UFMG), Belo Horizonte, MG, Brazil; 2 Department of Occupational Therapy, College of Health and Rehabilitation Sciences: Sargent College, Boston University, Boston, USA; 3 Departamento de Terapia Ocupacional de Lagarto, Universidade Federal de Sergipe (UFS), Lagarto, SE, Brazil

**Keywords:** assessment, functioning, translation, cultural adaptation, psychometric properties, rehabilitation

## Abstract

**Background:**

The Pediatric Evaluation of Disability Inventory-Computer Adaptive Test (PEDI-CAT), developed with innovative measurement methodologies, evaluates functioning of children and youth, from 0 to 21 years, with different health conditions. It is a revision of an earlier instrument (PEDI) that has been used in national and international clinical practice and research. It was felt to be necessary to make this new version (PEDI-CAT) available in Brazil.

**Objectives:**

Translate and culturally adapt the PEDI-CAT to the Brazilian-Portuguese language and test its psychometric properties.

**Method:**

This methodological study was developed through the following stages: (1) translation, (2) synthesis, (3) back-translation, (4) revision by an expert committee, (5) testing of the pre-final version, and (6) evaluation of the psychometric properties. The 276 translated PEDI-CAT items were divided into three age groups (0-7, 8-14, and 15-21 years).

**Results:**

The PEDI-CAT translation followed all six stages. The adaptations incorporated cultural and socioeconomic class specificities. The PEDI-CAT/Brazil showed good indices of inter-examiner (intraclass correlation coefficient-ICC=0.83-0.89) and test-retest (ICC=0.96-0.97) reliability, good internal consistency (0.99) and small standard error of measurement in all three age groups (0.12-0.17). Factor analyses grouped the items from the three functional skills domains into one factor, and items from the responsibility scale into three factors, supporting the adequacy of these factor solutions to the conceptual structure of the instrument and the developmental model.

**Conclusion:**

The PEDI-CAT/Brazil is a theoretically consistent, culturally appropriate, and reliable instrument. Its availability in Brazil will contribute to the evaluation and measurement of functional outcomes from clinical interventions, longitudinal follow-up, and rehabilitation research.

## BULLET POINTS

- PEDI-CAT item translation incorporated Brazilian-specific cultural adaptations.
- PEDI-CAT/Brazil has strong reliability, internal consistency and small SEM estimates.
- Factor solutions support its content adequacy to proposed conceptual structure.

## Introduction

Procedures for the translation and cultural adaptation of foreign functional measures are applied in Brazil to provide professionals and services with standardized instruments that are often used in other countries and cited in the international literature^1^ ^-^ ^8^. Because this is a complex and expensive process, with many structured stages and procedures^9^ ^-^ ^12^, it needs to be preceded by two important questions: (1) Is the instrument to be translated into another language relevant to clinical practice and scientific research? (2) Does the instrument have appropriate content and format for use in the country for which it is being made available?^13^.

The Pediatric Evaluation of Disability Inventory-Computer Adaptive Test (PEDI-CAT) affirmatively answers these two questions. This is the new version of the Pediatric Evaluation of Disability Inventory (PEDI). It incorporates innovative measurement methodologies, substantially extends the age range, and offers new items and a new format for the functional evaluation of children and youth, from 0 to 21 years of age, with various health conditions^14^.

The PEDI-CAT is a functional assessment that is theoretically grouded on the International Classification of Functioning (ICF), Disability and Health^15^ and the ICF-Children and Youth (ICF-CY)^16^. Based on the biopsychosocial and developmental models, it incorporates the sociocultural perspective^17^. In the biopsychosocial model, functioning reflects the interaction between individuals with a health condition and the opportunities or barriers present in the setting in which they live, including internal (personal) and external (environmental) factors. This interaction has a bidirectional influence on body structures and functions, activities and participation, which together represent the components of function. Based on the ICF-CY model, the PEDI-CAT contents provide information about the activities and participation component. In a socio-cultural approach, child's learning process of daily activities is grounded in guided participation and the transfer of responsibilities. Engagement of the child (i.e., the apprentice) with his/her parents and family members (i.e., experts) in the daily routine provides a context that triggers a collaborative process in which the child is guided by the expert to become engaged and gradually take responsibility for the performance of activities and tasks, while the caregiver decreases the assistance given^17^ ^,^ ^18^. This approach is used to guide the content and scoring criteria of the items on one of the PEDI-CAT test scales (i.e., Responsibility).

The theoretical-methodological foundation that grounded the construction of the PEDI-CAT was derived from the Rasch model. This is a probabilistic model, from Item Response Theory (IRT), which transforms ordinal information into interval measures^19^. In this model, individuals and items are calibrated hierarchically, in order of relative difficulty, making it possible to identify more and less difficult items as well as more and less skilled subjects on the same one-dimensional continuum, based on the calibrated items and subjects (logits)^20^. The computer adaptive testing method uses an algorithm to select the (calibrated) items to be administered while evaluating a particular individual in real time. For example, if the individual obtains a high score on the initial item, a new, more challenging item (i.e., one with greater relative difficulty) is displayed next, and so on, to obtain an accurate estimate of the individual's score along the functional continuum^14^. This technology results in less administration time, as there is no need to respond to all items on a scale.

Strong levels of reliability and validity for the PEDI-CAT^21^ are documented in the literature, and the instrument is used internationally in clinical practice and research^22^ ^,^ ^23^. In Brazil, the PEDI in its original version, is an outcome measure widely used in rehabilitation centers and clinical research. The availability of this new version (PEDI-CAT) will be very useful for professionals and managers of rehabilitation services, who will be able to identify the functional level of a child or youth, document changes occurring as a result of services/interventions, and accurately guide specific targets for functional treatment with greater time efficiency.

This study describes the procedures used for translation and cultural adaptation of the PEDI-CAT into Brazilian Portuguese and tested its psychometric properties (i.e. reliability and validity).

## Method

### PEDI-CAT description

The PEDI-CAT^24^ consists of four domains: (1) Daily Activities (DA), (2) Mobility (MB), (3) Social/Cognitive (SC), and (4) Responsibility (RS). It aims to provide a detailed description of the individual's function and document individual changes and the progress of functional skills acquired after an intervention. The PEDI-CAT is not a performance-based “test”, but rather, it consists of a large item bank of 276 functional activities acquired during childhood, adolescence, and early adulthood. Its application requires a computer with the instrument’s software installed and can either be self-administered (i.e. filled out by the child's parents), or a professional may be present with the parents to ensure understanding of the information for each item^14^.

In the DA, MB, and SC domains, scoring is based on a four-point ordinal scale with different levels of difficulty. The RS domain scores the items on a five-point scale, describing the sharing of responsibility between caregiver and child/youth in performing each item (Table 1). For the four domains, the respondent is asked to choose the option that best describes the child's function for each item. If the respondent is unsure, there is an option for answering, “I do not know”^14^.

**Table 1:** A brief description of the content of each domain and the scoring scale of the PEDI-CAT items.

**DOMAIN**	**CONTENT** ^*^	**ITEM SCORING SCALE**
**Daily Activities**	Getting Dressed, Keeping Clean, Home Tasks, and Eating and Mealtime	1) Unable = Cannot do, does not know how or is too young. 2) Hard = Performs with much help, extra time, or effort. 3) A little hard = Performs with a little help, extra time, or effort. 4) Easy = Performs with no help, extra time, or effort or the child’s skills are past this level. I Don’t Know.
**Mobility**	Basic Movement and Transfers, Standing and Walking, Steps and Inclines, Running and Playing, and Wheelchair	
**Social/Cognitive**	Interaction, Communication, Everyday Cognition, and Self Management	
**Responsibility**	Organization and Planning, Taking Care of Daily Needs, Health Management, and Staying Safe	1) Adult/caregiver has full responsibility; the child does not take any responsibility. 2) Adult/caregiver has most responsibility, and the child takes a little responsibility. 3) Adult/caregiver and child share responsibility approximately equally. 4) Child has most responsibility with a little direction, supervision, or guidance from an adult/caregiver. 5) Child takes full responsibility without any direction, supervision, or guidance from an adult/caregiver. I Don’t Know

* Information available at: http://pedicat.com/category/domains/.

### Translation and cultural adaptation

The PEDI-CAT translation process followed the methodology proposed by specific guidelines^9^ ^-^ ^11^ for this type of study, taking into consideration information from the PEDI-CAT translation guide sent by the authors after receiving authorization for the translation. This study was approved by the Research Ethics Committee of the Universidade Federal de Minas Gerais (UFMG), Belo Horizonte, MG, Brazil (CAAE: 20466614.0.0000.5149).

The translation and cultural adaptation followed six stages: (1) translation (2) synthesis, (3) back translation, (4) review by an expert committee, (5) testing of the pre-final version, and (6) testing of the psychometric properties.

Three pairs of independent bilingual translators, whose native language was Portuguese but who were fluent in English, translated the original English version of the PEDI-CAT items into Portuguese. The translators worked in independent pairs to foster the use of best consensus terminology in each translated version. They were either physical therapists or occupational therapists with significant experience in child development. This process resulted in three translated versions: T1, T2, and T3, which were analyzed in detail at a meeting with the translators and coordinators, resulting in a synthesized translated version, T123.

In the next stage, the T123 version was back translated into English independently by three other bilingual translators (BT1, BT2, and BT3) who had no knowledge of the original version of the instrument and who were not involved in the previous stage. The translators were familiar with English and Portuguese, and one was an English teacher.

In the fourth stage, an expert committee reviewed the versions (original, T123, BT1, BT2, and BT3) and discussed each item, searching for the best solution to solve the discrepancies and different alternatives in translation. Rather than focusing on indices of agreement, the committee attempted to make the best use of the language expertise of its members, solving the following types of disagreements: conceptual (referring to the conceptualization of the evaluated phenomenon), idiomatic (different linguistic expressions), semantic (differences related to the test content), and experiential (related to cultural differences). The expert committee was composed of two physical therapists and four occupational therapists who had not participated in the previous stages. One of these participants had extensive experience in psychometrics, one was the author of the original PEDI-CAT, and another was also an English teacher. The other three experts had participated in the translation and/or back-translation of other functional instruments. At this stage, additional reviews were conducted by professional from two rehabilitation centers in different regions of Brazil (i.e., southeast and northeast). These reviews aimed to determine whether the functional activities and the language used in the wording of the translated items were consistent with regional idiosyncrasies and could easily be understood by health professionals of various backgrounds^13^. Professionals from the Associação Mineira de Reabilitação (AMR) in Belo Horizonte, MG, and the Early Treatment and Stimulation Center (Núcleo de Tratamento e Estimulação Precoce-NUTEP) in Fortaleza, CE, Brazil contributed to the reviews. This judicious review process generated the pre-final version of the instrument, hereafter called the PEDI-CAT/Brazil.

### Testing the pre-final version

#### Participants

The pre-final version of the PEDI-CAT/Brazil was completed by a sample of 810 parents and/or guardians of children and youth from 0 to 21 years of age with normal development. Participants were recruited by convenience, informed about the study objectives, and asked to sign an informed consent form.

#### Procedures

Following the authors’ instructions, the 276 PEDI-CAT/Brazil items were distributed to the three age groups (0-7, 8-14, and 15-21 years of age) by experienced professionals in child development based on the age appropriateness of each item. These professionals did not include the translators or the panel of experts. It is important to stress that this division of items by age group was performed for the sole purpose of facilitating the subsequent collection of normative data, and the final Brazilian version of the instrument comprised all of the items related to all ages covered by the instrument.

After the three age-group versions were created, 20 administrators received detailed training on instrument content and data collection procedures. The standardized training lasted 30 hours. It included the following steps: (1) explanation of the instrument goals, (2) detailed explanation of each item and its scoring scale, (3) review of the use of the Economic Classification Criteria Brazil 2012, proposed by the Brazilian Association of Research Companies (Associação Brasileira de Empresas de Pesquisa-ABEP)^25^ to characterize the socioeconomic status of respondents, (4) scoring of training videos for all three age groups, and (5) evaluation of inter-examiner and test-retest reliability. After completing all of these steps, the administrators were deemed fit to administer the instrument.

The PEDI-CAT/Brazil was administered at a date, time, and place most convenient for the respondent. An additional strategy regarding the items’ scoring scales was adopted for all administrations. A supplement with the criteria from each response option from the three functional skills domains as well as from the responsibility scale was printed on cards that remained with the respondents while they completed the assessment. However, the score values (i.e., 1, 2, 3, and 4) were excluded from the cards to avoid influencing each respondent's answer. This method helped each respondent remember the response options for each item and thus facilitate and standardize data collection.

After completing the PEDI-CAT/Brazil, each respondent was asked to evaluate the adequacy of the instrument translation by answering the following questions: (1) What is your opinion of the translation of the PEDI-CAT? (2) Do the tasks listed in this questionnaire describe your child's function?^11^. According to the guidelines adopted in this study, a negative response percentage above 15% would indicate the need to revise the instrument's translation^26^ ^,^ ^27^.

#### Psychometric properties

The psychometric and measurement properties evaluated included inter-examiner and test-retest reliability, internal consistency, standard error of measurement (SEM) and factor analysis. Reliability estimates were calculated for each age group and for the 20 administrators, totaling 60 measures. The test-retest reliability evaluated the consistency of parents’ responses across two occasions when the PEDI-CAT items were administered with an interval of 7 to 15 days between the two tests. The inter-examiner reliability was evaluated by the stability of information when groups of examiners administered the PEDI-CAT items to the same parents. Internal consistency was examined using Cronbach's alpha, and a reference value above 0.70 was considered acceptable^28^. Data from the reliability analyses were used to calculate the SEM. Exploratory factor analysis (EFA) evaluated the organization of items from the three functional skills domains (i.e., Daily Activities, Mobility and Social/Cogntive ) and from the Responsibility domain into dimensions or concepts (i.e., factors) as well as the explanatory value of the factors solutions.

#### Statistical analyses

The participants were characterized using descriptive statistics. Intra-class correlation coefficients (ICC) and confidence intervals (CI) of the mean coefficients (95%) informed the inter-examiner and test-retest reliability. The criteria adopted for the ICC interpretation was: ICC<0.40-weak agreement; ICC≤0.75-moderate agreement and ICC> 0.75 indicated excellent agreement^29^. The SEM was calculated as the square root of the mean square error obtained in the analysis of variance^30^. Factor analysis adequacy was tested using the Kaiser-Meyer-Olkin (KMO) test and Bartlett's test of sphericity. Correlations above 0.50 in the KMO test and values of p <0.05 in Bartlett's test of sphericity indicated adequacy of the data for factor analysis. Measures of central tendency (i.e., mean) and variability (i.e., standard deviation [SD], variance, and covariance) were used to define the factor loadings and the eigenvalues assigned to the items. To define the number of factors in the final solution, the Kaiser Rule (i.e., eigenvalues above one) was used in addition to Scree plot analysis. Oblique rotation method (i.e., Oblimin rotation) optimized interpretation, as correlation among factors was assumed^31^.

The statistical analyses used the Statistical Package for the Social Sciences (SPSS), version 19.0.

## Results

### PEDI-CAT translation and cultural adaptation

Some differences in the versions analyzed by the expert committee during the PEDI-CAT translation process were observed and resolved using strategies such as addition, omission, or substitution of words and the provision of examples, in an attempt to reach semantic, conceptual, idiomatic, and experiential equivalence. The type and frequency of strategies adopted in each test domain are described in Table 2. Table 3 shows some examples of discrepancies found during the translation process and the strategies used by the expert committee to solve them.

**Table 2:** Number and type of strategies recommended by the Expert Committee in each domain of the PEDI-CAT.

**PEDI-CAT DOMAIN** ^*^	**PEDI-CAT ITEMS**	**STRATEGIES USED IN THE TRANSLATION** ^**^					**TOTAL**
		**EX**	**AD**	**OM**	**SUB**	**EXC**	
DA	68	7	5	4	0	0	**16**
MB	97	0	9	5	1	1	**16**
SC	60	0	5	0	1	0	**6**
RS	51	7	3	0	3	0	**13**
**TOTAL**	**276**	**14**	**22**	**9**	**5**	**1**	**51**

* Domains: DA: Daily Activities; MB: Mobility; SC: Social/Cognitive; RS: Responsibility.

** Strategies: EX: example; AD: addition, OM: omission; SUB: substitution; EXC: exclusion.

**Table 3:** Examples of translation strategies proposed by the Expert Committee.

**ITEM**	**ORIGINAL VERSION**	**TRANSLATED VERSION**		**STRATEGY**
DA013	Pours liquid from a large carton into a glass	Despeja o líquido de uma caixa **(por exemplo, de suco ou leite)** ^*^ em um copo	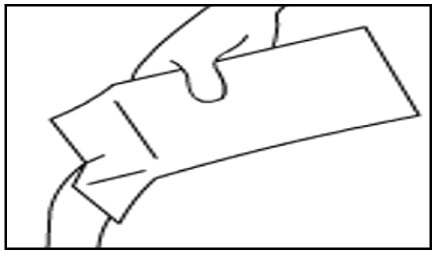	Example
DA021	Cuts with scissors to open hard plastic packaging	Abre uma embalagem de plástico duro (por exemplo, de brinquedo ou eletrônico)* usando tesoura	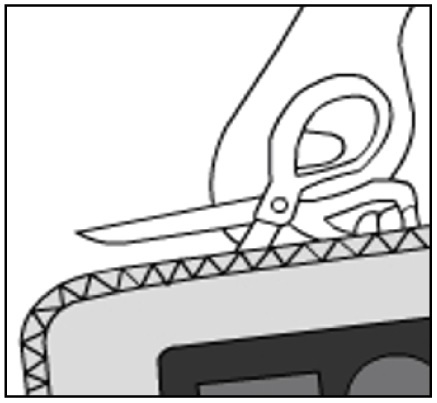	Example
MB095	Climbs on and off a climbing structure	Sobe e desce um brinquedo de escalar **(trepa-trepa)** ^*^	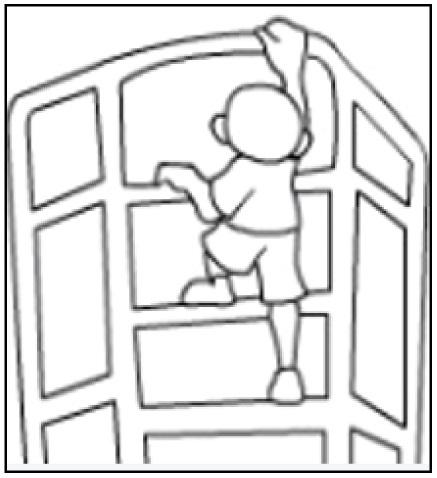	Addition
MB128	Walks 3 miles*/5 kilometers	Caminha por 5 quilômetros	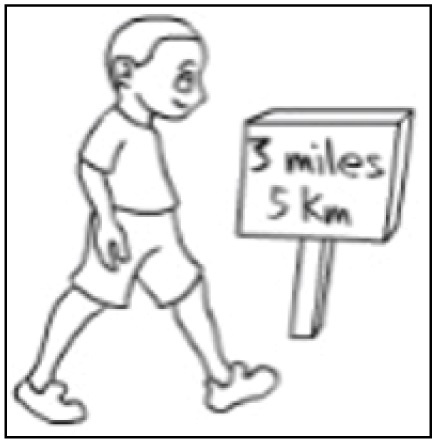	Omission
SC019	Plays **peek-a-boo** or **pat-a-cake** ^*^	Brinca de **“achou”** ou **jogos simples de bater palmas com as mãos** ^*^		Substitution
RS015	Following a recipe or cooking instructions that includes 3-4 ingredients and steps, such as macaroni and cheese or brownies	Segue uma receita ou instruções de culinária que incluem 3-4 ingredientes e passos, tais como **massa de bolo pronta, miojo, tapioca** ^*^		Substitution
RS055	Identifying correct polling location; Understanding the voting process and rights; Requesting absentee ballots as needed	Identificar o local correto de votação; Compreender o processo e os direitos/deveres de votar; Requisitar formulário de justificativa quando necessário; **Compreender os procedimentos necessários de uso da urna eletrônica** ^*^		Addition

* Parts in bold illustrate what was added, substituted and/or omitted in relation to the orginal item.

### Testing the pre-final PEDI-CAT/Brazil version

The pre-final PEDI-CAT/Brazil version was administered to the three age groups, with the greatest proportion in the 0 to 7-year age group (45%, n=367). The mean age was 9.37 years (SD=5.93), there was a greater percentage of females (57%), and individuals classified as socioeconomic level B (43%). Most respondents reported having no trouble understanding the items (95%) and that the content of the PEDI-CAT/Brazil adequately represented the child’s function in the family's daily life (98%) (Table 4).

**Table 4:** Testing of the pre-final version of the PEDI-CAT/Brazil.

**Sample Description and Information** **provided about the PEDI-CAT**			**0 to 7 years (n=367)**	**8 to 14 years (n=240)**	**15 to 21 years (n=203)**	**TOTAL (n=810)**
**Children/ Youth**	**Age** ^*^		3.78 (2.21)	11.17 (1.90)	17.36 (1.65)	9.37 (5.93)
	**Sex** ^**^	**F**	205 (56%)	131 (55%)	128 (63%)	464 (57%)
		**M**	162 (44%)	109 (45%)	75 (37%)	346 (43%)
**Family socioeconomic information** ^***^	**Socioeconomic classification**	**A**	52 (14%)	27 (11%)	31 (15%)	110 (14%)
		**B**	169 (46%)	94 (39%)	87 (43%)	350 (43%)
		**C**	122 (33%)	96 (40%)	73 (36%)	291 (36%)
		**D**	24 (7%)	23 (10%)	12 (6%)	59 (7%)
**About the PEDI-CAT**	**Difficulty demonstrated in understanding items**	**None**	342 (93%)	233 (97%)	193 (95%)	768 (95%)
		**A little**	22 (6%)	7 (3%)	10 (5%)	39 (5%)
		**A lot**	3 (1%)	0 (0%)	0 (0%)	3 (0%)
	**Do items represent functioning?**	**Yes**	359 (98%)	237 (99%)	195 (96%)	791 (98%)
		**No**	8 (2%)	3 (1%)	8 (4%)	19 (2%)

* Age: mean value (standard deviation).

** Sex: F: Female, M: Male, the values determine the frequency (percentage).

*** Family socioeconomic classification. The categories represent socioeconomic levels. They are defined from a standardized questionnaire that assigns points to items related to the presence and amount of certain home appliances, number of cars owned, and the level of formal education of the main family member (provider). The points are summed, and specific ranges are translated into categories, in which higher total scores refer to higher socioeconomic levels. Level A=46 to 35 points; B=23 to 34 points; C=14 to 22 points; and D=8 to 13 points.

### PEDI-CAT/Brazil reliability, internal consistency and SEM

The inter-examiner and test-retest reliabilities, internal consistency, and SEM were good to excellent, as shown in Table 5.

**Table 5:** Inter-examiner and test-retest reliability indices, internal consistency, and standard error of measurement values of the PEDI-CAT/Brazil.

	**0-7 years**	**8-14 years**	**15-21 years**
**Inter-examiner Reliability** **(95% CI)**	0.86 (0.81-0.90)	0.83 (0.76-0.88)	0.89 (0.85-0.92)
**Test-retest** **Reliability** **(95% CI)**	0.96 (0.95-0.97)	0.97 (0.97-0.98)	0.97 (0.96-0.98)
**Internal** **Consistency**	0.99	0.99	0.99
**Standard error of measurement**	0.12	0.17	0.13

The values reported in this table represent the lower reliability indices obtained from the analyses.

### Factor analysis

The KMO test showed correlations higher than 0.50, and the results of Bartlett's test of sphericity indicated p-values lower than 0.05, which indicate adequacy of the data for factor analysis. Initially, factor analysis of the items of the three functional skills domains identified four factors with eigenvalues greater than one. However, plot analysis showed that after the first factor, the variance curve became horizontal. Data were further analyzed by forcing solutions of three, two and one factor. In the three factors solution only one item (MB079-*Walks down a flight of stairs without holding onto handrail*) loaded on the second factor and three items (DA039-*Fastens hairclips or barrettes*, DA040-*Puts hair up in a ponytail* and DA074-*Puts on bra and fastens in front or back*) on the third factor. For the two factors solution, only one item (MB079) loaded on the second factor. Those analyses confirmed the plot information. Thus, the one factor solution best represented the conceptual (i.e. latent) structure underlying these items, explaining 89% of the variance.

Factor analysis of the Responsibility domain grouped items into three factors. Responsibility items from the 15 to 21-year age group were grouped into the first factor. Items from the 0 to 7 year and 8 to 14-year groups loaded on the second and third factors, respectively. This three factors solution explained approximately 82% of the item variance.

## Discussion

The translation and cultural adaptation of measurement instruments utilizes standardized methods and judicious criteria to develop appropriate versions of foreign instruments for use in countries with different languages^13^. This study rigorously followed the stages of internationally recognized guidelines^9^ ^-^ ^11^, resulting in the development of a translated version of the PEDI-CAT that incorporates the specifics of Brazilian culture and reality. It could be understood by health professionals and by parents of children and youth from different socioeconomic classes. In addition, procedures included a review stage of the translated version by professionals from rehabilitation centers in the southeast and northeast regions of the country.

Strategies such as word addition, omission, substitution and provision of examples were used to account for the varied socio-cultural and educational realities of the Brazilian population, while ensuring that the translated words and expressions remained faithful to each specific situation measured by the original instrument^6^. An example illustrates the translation of items from the Mobility domain. For item MB012 “*Sits on floor with pillow for support*”, it was necessary to determine whether it referred to the child in a static position or dynamically positioning him/herself. Thus, the term “*sits*” was translated as “*remains seated*” rather than “*sits*”*,* as the latter refers to the action of sitting and does not reflect a true translation of the situation measured by the original item.

Producing a valid translation into a language other than the one in which the original version was developed often presents difficult challenges, such as experiential differences between cultures. The expert committee involved in the development of the PEDI-CAT/Brazil version decided to remove item MB032 “*gets in and out of the bathtub*” and to omit or replace the terms “*bath*” and “*tub*” from items DA051 “*Cleans up thoroughly in bath or shower*” and RS020 “*Cleaning spills and wiping up food crumbs*; *Scrubbing sink and tub*; *Emptying trash*; *Replacing or repairing broken fixtures or objects*” because bath tubs are not common in Brazil. This adaptation had the consent of the PEDI-CAT authors, without prejudice to the functional evaluation. Previous Brazilian adaptation of the content of the original PEDI contributed to the development of items for the PEDI-CAT. The mobility item from the Portuguese version^32^ MB033 “*gets in and out of the shower*” was incorporated as an item in the new PEDI-CAT version.

A necessary precaution in the translation and cultural adaptation of instruments into Portuguese is the appropriateness of the language to the characteristics and peculiarities of different Brazilian regions. Given the extensive size of the country, cultural and linguistic differences among Brazilian regions should be considered in a translated instrument that will be used across the country^13^. In item RS035 “*Following a recipe or cooking instructions that includes 3 to 4 ingredients and steps such as macaroni and cheese or brownies*”, for example, the examples “*macaroni and cheese*” and “*brownies*” were replaced by *“massa de bolo pronta*” (i.e. cake mix), “*miojo*” (i.e. ramen noodles), and “*tapioca*”; the latter is a typical snack in the northern and northeastern regions (Table 3).

The pre-final version revealed that this PEDI-CAT/Brazil version was understood and evaluated as an adequate measure of function by most participants. Only 5% of respondents reported having difficulty understanding some items. It is only necessary to review a translation when the doubt index exceeds 15%^26^. However, it was observed that the respondents who reported having some difficulty in understanding cited some items in common, especially in the Responsibility domain. This is a complex domain that measures the transfer of responsibility from parents or other caregivers to children that occurs during learning relationships^18^. The items in this domain provide a detailed explanation of information that the respondent should consider for scoring. A possible strategy could, therefore, be to provide a small booklet in Portuguese containing very detailed criteria and detailed examples to assist in the choice of options for responses to some items of that domain. Such an initiative would require the approval of the original PEDI-CAT test authors.

High levels of reliability and internal consistency were found across all age groups in this study^29^. These results are similar to those reported for the original version^14^, supporting the PEDI-CAT/Brazil as a reliable instrument for the functional evaluation of Brazilian children and youth. The SEM values observed in the PEDI-CAT/Brazil, which reflect the degree of inaccuracy (i.e., measurement error) of the final score, were small (0.12 to 0.17 points). These values were also similar to those provided in the original PEDI-CAT manual^14^, suggesting that if the magnitude of a change in scores (e.g., after intervention or development) is higher than the SEM value, such a change should be attributed to the effect of changes resulting from the investigated process. Factor analysis showed that much of the variance in PEDI-CAT/Brazil items could be explained by one and three factors in the Functional Skills and Responsibility domains, respectively. The Responsibility items were grouped into factors related to age groups, indicating the suitability of the instrument for the development of children and youth’s responsibilities.

The collection of normative PEDI-CAT/Brazil data is the next stage of this project, which has the support of a second center in Fortaleza, CE, Brazil. The availability of a theoretically consistent functional instrument that is culturally appropriate and reliable, which requires a shorter administration time without losing the accuracy of estimates of the functional levels of infants, children, adolescents, and young adults, may contribute to improved evaluation and measurement of clinical intervention outcomes and longitudinal monitoring, further supporting advances in rehabilitation research.

## References

[1] Pilz B, Vasconcelos RA, Marcondes FB, Lodovichi SS, Mello W, Grossi DB (2014). The Brazilian version of STarT Back Screening Tool - translation, cross-cultural adaptation and reliability. Braz J Phys Ther.

[2] Lamarão AM, Costa LCM, Comper MLC, Padula RS (2014). Translation, cross-cultural adaptation to Brazilian-Portuguese and reliability analysis of the instrument Rapid Entire Body Assessment-REBA. Braz J Phys Ther.

[3] Furtado SRC, Sampaio RF, Vaz DV, Pinho BAS, Nascimento IO, Mancini MC (2014). Brazilian version of the instrument of environmental assessment Craig Hospital Inventory of Environmental Factors (CHIEF): translation, cross-cultural adaptation and reliability. Braz J Phys Ther.

[4] Lopes AR, Trelha CS (2013). Translation, cultural adaptation and evaluation of the psychometric properties of the Falls Risk Awareness Questionnaire (FRAQ): FRAQ-Brazil. Braz J Phys Ther.

[5] Pereira LM, Dias JM, Mazuquin BF, Castanhas LG, Menacho MO, Cardoso JR (2013). Translation, cross-cultural adaptation and analysis of the psychometric properties of the lower extremity functional scale (LEFS): LEFS- BRAZIL. Braz J Phys Ther.

[6] Amaral MF, Paula RL, Drummond A, Dunn L, Mancini MC (2012). Translation of the Children Helping Out: Responsibilities, Expectations and Supports (CHORES) questionnaire into Brazilian-Portuguese: semantic, idiomatic, conceptual and experiential equivalences and application in normal children and adolescents and in children with cerebral palsy. Braz J Phys Ther.

[7] Puga VOO, Lopes AD, Costa LOP (2012). Assessment of cross-cultural adaptations and measurement properties of self-report outcome measures relevant to shoulder disability in Portuguese: a systematic review. Braz J Phys Ther.

[8] Maher CG, Latimer J, Costa LOP (2007). The relevance of cross-cultural adaptation and clinimetrics for Physical Therapy instruments. Braz J Phys Ther.

[9] Beaton DE, Bombardier C, Guillemin F, Ferraz MB (2000). Guidelines for the process of cross-cultural adaptation of self-report measures. Spine (Phila Pa 1976).

[10] Guillemin F, Bombardier C, Beaton D (1993). Cross-cultural adaptation of health-related quality of life measures: literature review and proposed guidelines. J Clin Epidemiol.

[11] Van Widenfelt BM, Treffers PDA, Beurs E, Siebelink BM, Koudijs E (2005). Translation and cross-cultural adaptation of assessment instruments used in psychological research with children and families. Clin Child Fam Psychol Rev.

[12] Mokkink LB, Prinsen CA, Bouter LM, Vet HC, Terwee CB (2016). The COnsensus-based Standards for the selection of health Measurements INstruments (COSMIN) and how to select an outcome measure instrument. Braz J Phys Ther.

[13] Coster WJ, Mancini MC (2015). Recommendations for translations and cross-cultural adaptation of instruments for occupational therapy research and practice. Rev Ter Ocup Univ São Paulo.

[14] Haley SM, Coster WJ, Dumas HM, Fragala-Pinkham MA, Moed R (2012). PEDI-CAT: development, standardization and administration manual.

[15] World Health Organization – WHO (2001). International classification of functioning, disability and health.

[16] World Health Organization – WHO (2007). International classification of functioning, disability and health children and youth version.

[17] Rogoff B, Sameroff A, Haith MM (1996). Developmental transitions in children’s participation in sociocultural activities. The five to seven year shift: the age of reason and responsibility.

[18] Rogoff B (1990). Apprenticeship in thinking: cognitive development in social context.

[19] Ayala RJ (2009). The theory and practice of item response theory.

[20] Chang W, Chan C (1995). Rasch analysis for outcome measures: some methodological considerations. Arch Phys Med Rehabil.

[21] Dumas HM, Fragala-Pinkham MA, Haley SM, Ni P, Coster W, Kramer JM (2012). Computer adaptive test performance in children with and without disabilities: prospective field study of the PEDI-CAT. Disabil Rehabil.

[22] Kao YC, Kramer JM, Liljenquist K, Tian F, Coster WJ (2012). Comparing the functional performance of children and youths with autism, developmental disabilities, and no disability using the revised pediatric evaluation of disability inventory item banks. Am J Occup Ther.

[23] Kramer JM, Coster WJ, Kao YC, Snow A, Orsmond GI (2012). A new approach to the measurement of adaptive behavior: development of the PEDI-CAT for children and youth with autism spectrum disorders. Phys Occup Ther Pediatr.

[24] Pediatric Evaluation of Disability Inventory Computer Adaptive Test – PEDI-CAT (2015). Information about the PEDI-CAT (English version).

[25] Associação Brasileira de Empresas de Pesquisa – ABEP (2015). A ABEP: Critério Brasil [Brazilian Market Research Association].

[26] Ciconelli RM, Ferraz MB, Santos W, Meinão I, Quaresma MR (1999). Brazilian-Portuguese version of the SF-36: a reliable and valid quality of life outcome measure. Rev Bras Reumatol.

[27] Nusbaum L, Natour J, Ferraz MB, Goldenberg J (2001). Translation, adaptation and validation of the Roland-Morris questionnaire. Braz J Med Biol Res.

[28] Nunnally JC, Bernstein IH (1994). The assessment of reliability. Psychometric Theory.

[29] Portney LG, Watkins MP (2000). Foundations of clinical research: applications to practice.

[30] Lexell JE, Downham DY (2005). How to assess the reliability of measurements in rehabilitation. Am J Phys Med Rehabil.

[31] Hair JR, Black WC, Babin BJ, Anderson RE, Tatham RL (2006). Multivariate data analysis.

[32] Mancini MC (2005). Pediatric Evaluation of Disability Inventory (PEDI): manual of the Brazilian adapted version.

